# Oral health and oral health behavior in young adults with caries disease

**DOI:** 10.1038/s41405-021-00084-3

**Published:** 2021-07-31

**Authors:** Jennie Hagman, Ulla Wide, Helene Werner, Magnus Hakeberg

**Affiliations:** Department of Behavioral and Community Dentistry, Institute of Odontology, Sahlgrenska Academy, University of Gothenburg, Gothenburg, Sweden

**Keywords:** Gingivitis, Dental epidemiology

## Abstract

**Objective:**

The aim of this study was to describe and analyze oral health, oral health behaviors, and oral health-related quality of life (OHRQoL) in relation to the level of caries disease among caries-active young adults.

**Material and methods:**

This study presents data from a sample of young adults (*n* = 135) with active caries disease who were enrolled in a clinical, randomized controlled trial. The independent variables of sociodemographics, oral health (gingivitis, plaque), oral health behaviors (such as toothbrushing, dental attendance, sugar-containing sweets and drinks), dental anxiety, self-rated oral health, and OHRQoL were collected. Multinomial logistic regression was used to simultaneously evaluate the associations between the independent variables and caries severity.

**Results:**

Multinominal logistic regression showed that poor OHRQoL and gingivitis were associated with caries severity in a gradient fashion in accordance with caries disease activity. Also, irregular dental care and frequent consumption of sugary soda were significantly associated with very high caries severity.

**Conclusions:**

The risk factors related to caries severity among young adults were poor OHRQoL, gingivitis, consumption of sugary soda and irregular dental care attendance, indicating the need for a combination of different interventions specifically health behavior change. Furthermore, these findings may contribute to identifying high caries-risk individuals.

## Introduction

Dental caries in permanent dentition is one of the world’s most common diseases, affecting individuals throughout their lifetime.^1^ It may cause pain, discomfort, and anxiety and, if left untreated, lead to the spread of infection and tooth loss. This condition may not only affect an individual’s ability to eat and speak properly but may also result in lost work and school hours and affect the individual’s overall wellbeing.^2^ Dental caries is a multifactorial disease caused by both biological and behavioral factors, some of which (e.g., diet) are shared with other noncommunicable diseases (diabetes, heart disease, cancer).^1^

The caries prevalence has decreased substantially during the last decades, mainly in developed countries. In a repeated cross-sectional population survey among adults from 1983 to 2013, the caries prevalence dropped by 50%, and for 35-year-olds, the number of manifest caries lesions decreased from 2.0 to 0.9 over the 30-year time span.^3^ However, there are subgroups in populations that are affected by a high caries prevalence, which demonstrates the need for risk factor analysis as well as preventive and dental care interventions.

Several studies have been published concerning the associations between the effects of different risk factors (such as dental anxiety, oral health care habits, attitudes, socioeconomic status), over and above well-known etiological factors such as sugars and different types of bacteria with cariogenic properties, for the occurrence and distribution of oral diseases (i.e., dental caries).^4–11^ Little is known about the relationship between these risk factors and caries severity among young adults with a high disease burden. Identifying these key determinants of caries disease is important in the work to pursue more effective oral health promotion strategies in caries-active young adults, as well as finding high caries risk individuals in the clinic.

Furthermore, it is important to target young adults, as they are in the process of shaping their future adult health care habits.^12^ Establishing and maintaining positive oral health beliefs and confidence in the dental profession during early adulthood is crucial for the oral health outcome and oral health-related quality of life (OHRQoL), also later in life.^13,14^ A few reports have been published on dental caries and their association with OHRQoL in young adults. But the results are inconsistent, with some studies reporting an association,^15–18^ while others do not.^19–21^ Further studies of the relationship between caries disease and OHRQoL are therefore warranted.

According to Swedish law, the regions are obligated to provide dental care, free of charge, up to the age of 23 years, for all its inhabitants. Around 90% of all children, adolescents, and young adults attend the Public Dental Service clinics on a recall basis, based on both age and the risk of oral diseases. This makes the Public Dental Service (PDS) a suitable arena for research on oral health and oral health behavior among young adults.

The aim of this study was to describe and analyze oral health, oral health behaviors, and OHRQoL in relation to the level of caries disease among caries-active young adults.

## Material and methods

### Design

This study presents baseline data from a randomized controlled trial (RCT) (TRN ISRCTN15009620) that evaluated a psychological oral health intervention providing two cognitive behavior therapy sessions at dental clinics.^22^ All participants in the RCT and a selection of baseline variables were included in the present study.

The Declaration of Helsinki protocols were followed, and the study was approved by The Regional Ethical Board in Gothenburg (reg. no. 840-12).

### Participants

Recruitment of participants took place at two PDS clinics in Region Västra Götaland, Sweden, between 2013 and 2014. All young adults were screened for eligibility while attending their regular dental examination at their dentist or dental hygienist. Patients that met the inclusion criteria (18–25 years of age, and at least two manifest proximal dental caries lesions since the last dental examination) but not the exclusion criteria (psychiatric/neuropsychiatric diagnosis or not fluent in Swedish) were consecutively asked to participate. At the time of the recruitment of study participants, around ten dentists and six dental hygienists worked in each PDS clinic. Of 186 eligible individuals, 51 declined to participate, with the most common reasons being “not interested” (*n* = 24) and “lack of time” (*n* = 22). The remaining 135 individuals were included in the trial after they had given written informed consent. A more detailed description of the recruitment procedure has been published earlier.^22^

### Measurements

Questions about sociodemographic variables, self-rated oral health, oral health behaviors, oral health-related quality of life, and dental anxiety were answered by the participants on a touch-screen computer.

*Sociodemographic characteristics* were measured with questions about sex, age, ethnicity (born in a Nordic country [Swedish-born, other Nordic country] or other countries); occupation (employed/student or unemployed); parents’ country of birth (born in Nordic country [Swedish-born, other Nordic country] or other countries); parents’ education (primary school, secondary school, university).

*Self-rated oral health* was measured with the question: “How do you rate your oral health?”, with four response options (poor, fair, good, very good).

*Oral health behaviors* were captured with the questions “How often do you use a toothbrush?” and “How often do you use fluoridated toothpaste?”, with six response options, dichotomized into ≥ twice a day (three times a day or more - twice a day), or ≤ once a day (once a day, several times a week, once a week, never or seldom); “How often do you use additional fluorides?” and “How often do you use dental floss?”, with six response options, dichotomized into ≥ several times a week (three times a day or more, twice a day, once a day, several times a week), or ≤ once a week (once a week, never or seldom); “How often do you consume sugary sodas?” and “How often do you consume sweets?”, with six response options, dichotomized into often (several times a day, once a day, several times a week), or seldom (once a week, seldom, never); “How often do you attend dental care?” was measured with six response options, dichotomized into ≥ once a year (twice a year, once a year), or ≤ once every second year (once every second year, less then every second year, only for acute dental need, never). “Do you smoke?” was measured with the response options “yes” or “no”. The dichotomization of oral health behaviors was based on standard recommendations,^23,24^ while the variables sweets and sugary soda were dichotomized according to the midpoint of the scale used.

*Oral health-related quality of life* was assessed by the five-item version of the Oral Health Impact Profile (OHIP-5) questionnaire.^25^ It consists of five items addressing problems related to pain, oral function, oral psychosocial impact and appearance. Each item has five response alternatives (never (0), hardly ever, occasionally, fairly often, very often (4)), generating the sum score of 0-20, with a higher score indicating poorer OHRQoL. The OHIP-5 was analyzed as a sum score and as dichotomized into good OHRQoL (scoring ≤1 on all items) and poor OHRQoL (scoring ≥ 2 on at least one item).^17^

*Dental anxiety* was assessed by the four-item Dental Anxiety Scale (DAS).^26^ The items are scored 1–5, with sum scores of 4-20. A higher score indicates more dental anxiety.

*Gingivitis* was recorded at six index teeth (16, 21, 24, 44, 41, and 36),^27^ at the buccal, mesial, distal, and lingual/palatinal surfaces. Gingivitis was present when bleeding was recorded on probing the gingival sulcus.^28^ In the present analysis, gingivitis is presented as the sum of surfaces diagnosed with gingivitis (range 0–24).

*Plaque* was recorded on the same index teeth and surfaces as for gingivitis and registered according to the Silness-Löe plaque index system, with each surface given a score between 0 and 3.^29^ The plaque scores in the present analysis were dichotomized into “absence of visible plaque” (score 0-1) or “presence of visible plaque” (score 2–3), and presented as the sum of surfaces with visible plaque (range 0–24).

*Dental caries* lesions were registered for all tooth surfaces by clinical and radiographic (bitewing radiographs) examination. Each surface was recorded as initial or manifest caries.^30^ The sum of manifest caries lesions was calculated for each participant. Additionally, the sum score for manifest lesions was trichotomized, based on the percentiles 33 and 67, to create a moderate caries group (2–3 manifest lesions), a high caries group (4–6 manifest lesions), and a very high caries group (≥7 manifest lesions), in order to evaluate associations in relation to other variables.

Interexaminer reliability was assessed for the diagnosis of manifest caries lesions on bite-wing radiographs (*n* = 31) with a kappa value of 0.82, indicating very high agreement.^31^

### Statistical analyses

Mean, medians (Md), standard deviations (SD), and frequencies were calculated for the descriptive analysis. For group comparisons, the Chi-square test was applied to categorical variables. The Kruskal-Wallis test was used to evaluate any difference between the three caries groups for continuous variables and the Mann-Whitney test was applied for post-hoc analysis. Multinomial logistic regression analyses were performed for the dependent outcome variable of caries, trichotomized into moderate, high, and very high caries groups. In the statistical analyses, the independent variables were divided into five different models based on similarities among the variables: (i) consumption of sugars, (ii) oral health behaviors, (iii) OHRQoL and dental anxiety, (iv) objective oral health, and (v) socioeconomic indicators. The Likelihood ratio test was used to evaluate the significance of each model and variables for the inclusion in the final model. The entry procedure was applied for each model. *P* < 0.05 was chosen as level of significance.

## Results

### Sample characteristics

Of the 135 individuals participating in the study, 64 were women and 71 men, mean age 20.6 (SD = 2.2). The participants had a mean number of 5.6 (SD = 4.6) carious tooth surfaces and the proportion of tooth surfaces/gingival surfaces affected by plaque and gingivitis, were 32% and 68%, respectively. Furthermore, the participants reported a mean OHIP-5 score of 5.2 (SD = 3.3, Md = 5).

### Bivariate analyses

Oral health behavior (i.e., consumption of sugary sodas, dental care attendance), sociodemographic characteristics (i.e., father’s ethnicity), self-rated oral health, OHRQoL, dental anxiety, and clinical objective parameters (i.e., gingivitis, plaque levels) were found to differ significantly according to caries severity (Tables 1 and 2). In addition, the significant differences between caries groups showed a gradient feature with regard to caries severity.

**Table 1:** Sociodemographic characteristics, self-rated oral health, OHRQoL (measured by the OHIP-5), and dental anxiety (measured by the DAS) according to caries severity: moderate caries (2–3 lesions), high caries (4–6 lesions), very high caries (≥7 lesions), and for the total sample.

Variables *n* (%)	Moderate caries (*n* = 54)	High caries (*n* = 46)	Very high caries (*n* = 35)	*p* value	Total (*n* = 135)
Female	26 (48.1)	20 (43.5)	18 (51.4)	0.829	64 (47.4)
Smoker	13 (24.1)	19 (41.3)	15 (42.9)	0.052	47 (34.8)
Nordic-born	44 (81.5)	37 (80.4)	22 (62.9)	0.058	103 (76.3)
Mother Nordic-born	34 (63.0)	23 (50.0)	16 (45.7)	0.096	73 (54.1)
Father Nordic-born	34 (63.0)	24 (52.2)	14 (40.0)	0.034	72 (53.3)
Employed/student	42 (77.8)	39 (84.8)	23 (65.7)	0.266	104 (77.0)
Mother’s education					
Primary school	12 (22.2)	14 (30.4)	11 (31.4)	0.108	37 (27.4)
Secondary school	23 (42.6)	27 (58.7)	16 (45.7)		66 (48.9)
University studies	19 (35.2)	5 (10.9)	8 (22.9)		32 (23.7)
Father’s education					
Primary school	13 (24.1)	15 (32.6)	12 (34.3)	0.664	40 (29.6)
Secondary school	32 (59.3)	20 (43.5)	16 (45.7)		68 (50.4)
University studies	9 (16.7)	11 (23.9)	7 (20.0)		27 (20.0)
Self-rated oral health					
Poor	12 (22.2)	19 (41.3)	21 (60.0)	0.001	52 (38.5)
Fair	30 (55.6)	22 (47.8)	10 (28.6)		62 (45.9)
Good	12 (22.2)	5 (10.9)	4 (11.4)		21 (15.6)
Very good	0 (0.0)	0 (0.0)	0 (0.0)		0 (0.0)
OHIP-5, mean (SD)	4.5 (3.3)	5.6 (3.2)	5.9 (3.1)	0.057	5.2 (3.3)
Poor OHRQoL^a^	33 (61.1)	37 (80.4)	29 (82.9)	0.016	99 (73.3)
DAS, mean (SD)	7.7 (4.0)	7.8 (3.7)	9.8 (4.6)	0.049	8.3 (4.1)

The Chi-square test was applied to categorical variables.

The Kruskal-Wallis test was used for continuous variables.

The Mann-Whitney test was applied for post-hoc analysis of continuous variables.

^a^OHIP-5 dichotomized.

**Table 2:** Oral health behaviors and clinical parameters according to caries severity: moderate caries (2–3 lesions), high caries (4–6 lesions), very high caries (≥7 lesions), and for the total sample.

Variables *n* (%)	Moderate caries (*n* = 54)	High caries (*n* = 46)	Very high caries (*n* = 35)	*p* value	Total (*n* = 135)
Consuming sugary sodas often	24 (44.4)	26 (56.5)	27 (77.1)	0.003	77 (57.0)
Consuming sweets often	22 (40.7)	16 (34.8)	13 (37.1)	0.687	51 (37.8)
Toothbrushing ≥ twice a day	35 (64.8)	23 (50.0)	25 (71.4)	0.711	83 (61.5)
Use of toothpaste ≥ twice a day	35 (64.8)	25 (54.3)	24 (68.6)	0.856	84 (62.2)
Dental flossing ≥ several times a week	21 (38.9)	14 (30.4)	16 (45.7)	0.630	51 (37.8)
Use of additional fluorides ≥ several times a week	30 (55.6)	27 (58.7)	20 (57.1)	0.856	77 (57.0)
Dental care attendance ≥ once a year	48 (88.9)	42 (91.3)	24 (68.6)	0.019	114 (84.4)
Gingivitis, mean (SD)	14.2 (7.1)	17.0 (6.1)	19.0 (6.7)	0.002	16.4 (6.9)
Plaque, mean (SD)	5.9 (6.2)	8.8 (6.2)	8.5 (6.6)	0.022	7.6 (6.4)

The Chi-square test was applied to categorical variables. The Kruskal-Wallis test was used for continuous variables. The Mann-Whitney test was applied for post-hoc analysis of continuous variables.

Although no difference was shown between the three caries groups and the OHIP mean scores (*p* = 0.057), a difference was found between the moderate and the very high caries groups (*p* = 0.030).

### Multivariable analysis

In order to reveal associations between the independent variables and caries severity, five different statistical models that included theoretically important independent variables were analyzed. Based on these assumptions, four statistically significant models are shown in Table 3. The fifth model included socioeconomic indicators (sex, mother’s education) and was found non-significant and therefore not included in Table 3. The statistically significant variables in models 1–4, respectively, were then included in a final model to reveal the association of each variable to caries severity. Thus, the variables consumption of sugary soda, dental care attendance, OHRQoL, and gingivitis were applied in the final model (Table 4). Specifically, belonging to the very high caries group rather than the moderate caries group was more likely if consuming sugary soda often, rather than less often, as well as reporting irregular dental care, rather than regular dental care. Moreover, reporting poor OHRQoL rather than good, as well as having high levels of gingivitis, were found to be associated with the high and very high caries groups compared with the moderate group.

**Table 3:** Multinomial logistic regression, using caries group as the dependent variable, testing different models of sugar consumption, oral health behaviors, OHRQoL and dental anxiety, and oral health as the independent variables.

	Model 1 Sugar consumption (*p* = 0.007)			Model 2 Oral health behaviors (*p* = 0.015)			Model 3 OHRQoL & DA (*p* = 0.047)			Model 4 Oral health (*p* = 0.030)		
		OR	95% CI		OR	95% CI		OR	95% CI		OR	95% CI
High caries group	Sugary soda often	ref		Using toothpaste often	Ref.		Good OHRQoL	0.38	0.15-0.95	Gingivitis	1.03	0.96-1.11
	Sugary soda less often	0.60	0.27–1.34	Using toothpaste less often	0.64	0.29–1.44	Poor OHRQoL	Ref.				
	Sweets often	Ref.		Irregular dental care	0.75	0.20–2.85	DA	0.99	0.89–1.10	Plaque	1.06	0.98–1.14
	Sweets less often	0.49	0.33–1.70	Regular dental care	Ref.							
Very high caries group	Sugary soda often	Ref.		Using toothpaste often	Ref.		Good OHRQoL	0.36	0.13–1.04	Gingivitis	1.17	1.02–1.22
	Sugary soda less often	0.23	0.09–0.61	Using toothpaste less often	1.22	0.48–3.11	Poor OHRQoL	Ref.				
	Sweets often	Ref.		Irregular dental care	3.69	1.22–11.21	DA	1.11	0.99–1.24	Plaque	1.01	0.93–1.10
	Sweets less often	0.78	0.31–1.93	Regular dental care	Ref.							

*OR* Odds ratio, *95% CI*  95% Confidence intervals, *OHRQoL* Oral health-related quality of life, *DA* Dental anxiety, *Ref.* Reference category.

The moderate caries group is the reference group.

**Table 4:** Multinomial logistic regression using caries group as the dependent variable with the moderate caries group as the reference group.

	Final model		
		OR	95% CI
High caries group	Sugary soda often	Ref.	
	Sugary soda less often	0.81	0.35–1.88
	Irregular dental care	0.83	0.21–3.23
	Regular dental care	Ref.	
	Good OHRQoL	0.38	0.15–0.97
	Poor OHRQoL	Ref.	
	Gingivitis	1.06	1.00–1.13
Very high caries group	Sugary soda often	Ref.	
	Sugary soda less often	0.35	0.12–0.98
	Irregular dental care	4.13	1.20–14.22
	Regular dental care	Ref.	
	Good OHRQoL	0.37	0.12–1.17
	Poor OHRQoL	Ref.	
	Gingivitis	1.11	1.03–1.20

*OR* Odds ratio, *95% CI* 95% Confidence intervals, *OHRQoL* Oral health-related quality of life, *Ref.* Reference category.

Final model of included statistically significant independent variables.

## Discussion

This study showed a gradient association between caries severity and OHRQoL, sugar consumption, irregular dental care, and the amount of gingivitis among young adults with caries disease.

There are some limitations and strengths to this study that should be considered when interpreting the results. Firstly, the study lacks a healthy control group. Secondly, the cross-sectional study design implies that no causal links can be inferred. Thirdly, initial caries lesions were excluded from the analysis, thereby underestimating the actual caries prevalence in the sample and possibly also showing a weaker association between risk factors and the caries burden in the sample. Moreover, there are no data on oral health among the group of nonparticipants; hence, the severity of the caries burden in the sample may be underestimated. One strength of the study is that it consists of a fairly large clinical sample of young adults affected by a large number of manifest caries lesions. In addition, the sample comprised individuals attending general dental clinics at the PDS and not specialist/hospital clinics, which further strengthens the generalization perspective of the results. Furthermore, to our knowledge, there are no previous reports exploring oral health behavior, clinical status, OHRQoL and dental anxiety, and their associations with caries severity in a clinical sample of highly caries-active young adults.

In one study of Swedish 20-year olds, about 24% of the study sample had manifest caries lesions with a mean number of 0.5 surfaces with manifest caries lesion.^32^ In another Swedish epidemiological cross-sectional study, the number of caries-affected surfaces (including both manifest and initial lesions) was 3.7 among 20-year-old study participants.^33^ In the present study, the mean number of dental surfaces with manifest caries lesion was 5.6, indicating that the study participants suffered from a high caries disease burden.

Previous research associated with caries experience in young adults lists risk factors such as socioeconomic and demographic factors, toothbrushing frequency, and dental anxiety.^4–6,34–36^ The present study did not find similar associations in the logistic regression analysis. In fact, there were four other factors that were significantly associated with higher levels of caries disease: gingivitis, OHRQoL, sugary soda consumption, and dental attendance behavior. Moreover, the present sample had a much higher proportion of negative oral health behaviors compared with the general population of adults, with respect to toothbrushing frequency and consumption of sugary sodas.^37^ For example, in the present sample, a high consumption of sugary sodas and less frequent toothbrushing were reported by 57.0% and 38.5%, respectively. The corresponding figures for one Swedish study revealed 26% and 16%, respectively, in a general population.^37^ Furthermore, in a population study of 19–20-year-old Norwegians, 17.5% of the respondents reported that they consumed sugary sodas at least daily.^38^

Sociodemographic variables, such as migration background, parents born abroad, and parental educational level, are well-known risk factors for dental caries; however, only father’s ethnicity was found to be statistically significantly associated with the caries level in the present sample. Nevertheless, the association between these risk factors and dental caries were demonstrated in part, as the parents of the present study participants had a lower educational level in general and a greater proportion were born outside the Nordic countries, compared with the general population of region Västra Götaland at the time of the study.^39^

The vast majority of the study participants visited the dental clinic at least once a year; however, 15.6% visited only once every second year or even less frequent. In addition, the group of study participants that suffered from the highest burden of caries disease also had the greatest proportion of study participants that received dental care irregularly (31.4%). Although the reason for this is not investigated in the present study, the results also demonstrated that this group reported the highest dental anxiety. There are several studies in the literature that have found that patients suffering from dental anxiety cancel/miss appointments more frequently and experience poorer oral health than a general population.^40,41^ This may be an explanation for the less frequent dental care attendance reported in this group. Another possible reason for not visiting the dental service every year may be misclassification by the dentists or dental hygienists regarding the caries risk among some of the study participants, resulting in longer re-call periods.

The level of dental anxiety in the present study is similar to the levels in previous reports about young adults, such as population-based data for 26-year-old New Zealanders^42^ and 25-year-old Norwegians.^43^ However, the participants belonging to the very high caries group reported a statistically significantly higher mean score on the DAS compared with the moderate and high caries group, but the mean score did not reach the frequently used cut-off point for dental anxiety (score of ≥13).^44^

To our knowledge, the OHIP-5 has not previously been used to measure OHRQoL in a young adult population affected by severe caries disease. However, the median value in the present sample (Md = 5) is clearly higher than the normative values of the Swedish population (Md = 1), including the subpopulation of edentulous individuals and wearers of complete dentures (Md = 2).^45^ Even if the results from this study may not be directly comparable to those of other studies, due to factors such as different culture, age range, OHIP version, and study setting/design, it is evident that the young adults in the present study suffer from poor OHRQoL. The present study revealed an association between the caries burden and poor OHRQoL, which is in line with previous findings by Lawrence et al.,^46^ but in contrast to Flink et al.^47^ The proportion of the present sample reporting poor OHRQoL also seems to be very high (70%), in contrast to findings in other studies (50%).^17,48^

## Conclusions

A higher burden of caries disease was associated with poorer oral health-related quality of life, more gingivitis, and higher consumption of sugar-containing sodas, and less frequent dental care attendance. It is obvious, based on these results, that despite the fact that all children in Sweden are offered full regular dental care free-of-charge up to young adulthood with a focus on prevention, young adults with severe caries disease still display a combination of different known risk factors with regard to health behaviors and perceived oral health-related quality of life. The findings may also contribute to identifying high caries-risk individuals. These individuals require special attention and care in the clinic. The dental profession may also need better tools in the clinic and collaboration with other professionals, such as dieticians and psychologists, in order to help individuals with severe caries disease.
